# Intracellular Protein Binding of Zr-89 Oxine Cell Labeling for PET Cell Tracking Studies

**DOI:** 10.3390/pharmaceutics17040518

**Published:** 2025-04-15

**Authors:** Emmanuel Nyong, Yutaka Kurebayashi, Kingsley O. Asiedu, Peter L. Choyke, Noriko Sato

**Affiliations:** 1 Molecular Imaging Branch, Center for Cancer Research, National Cancer Institute, National Institutes of Health, Bethesda, MD 20892, USA; ecnyong@utmb.edu (E.N.); y_kurebayashi@keio.jp (Y.K.); kingsley.asiedu@duke.edu (K.O.A.); pchoyke@mail.nih.gov (P.L.C.); 2 Department of Surgery, The University of Texas Medical Branch, Galveston, TX 77555, USA; 3 Department of Pathology, Keio University School of Medicine, Tokyo 160-8582, Japan; 4 Department of Radiology, Duke University Medical Center, Durham, NC 27710, USA

**Keywords:** zirconium-89 oxine, cell labeling, protein labeling, cell tracking, positron emission tomography, radiolabeling

## Abstract

**Background/Objectives**: ^89^Zr-oxine is an ex vivo cell labeling agent that enables cells to be tracked in vivo by positron emission tomography (PET) over a period of up to two weeks. To better understand where ^89^Zr-oxine binds within cellular components, factors affecting labeling and intracellular distribution of ^89^Zr were examined. **Methods**: Mouse primary T cells, natural killer cells, dendritic cells, and monocytes, and cell lines EL4 (mouse lymphoma), DC2.4 (mouse dendritic cell), Kit225K6 (human T cell leukemia) and MC38 (mouse colon adenocarcinoma) were labeled with ^89^Zr-oxine or ^111^In-oxine and protein binding within the cellular compartments, the labeling thresholds, and radioactivity retention were subsequently determined. **Results**: Cell incorporation of ^89^Zr-oxine (27.8–71.8 kBq/10^6^ cells) positively correlated with cellular size and protein mass. Most (>97%) ^89^Zr was protein-bound and primarily localized in the cytoplasm, membrane, and nuclear fractions (>81%) with distribution patterns varying by cell type. By contrast, ^111^In-oxine showed lower protein-binding activity of approximately 59–65%, with 62–65% of ^111^In localized in the cytoplasm. Autoradiography of electrophoresed subcellular fractionated cell samples indicated stable binding by ^89^Zr-oxine to proteins in all subcellular fractions but unstable protein binding by ^111^In. Saturation studies showed that ^89^Zr-oxine labeling was saturable, and further labeling reduced cellular retention. Biodistribution of dendritic cells labeled with either ^89^Zr-oxine or ^111^In-oxine indicated greater retention of ^89^Zr in the labeled cells in vivo than ^111^In. **Conclusions**: ^89^Zr-oxine stably binds many intracellular proteins and shows much higher and more stable protein binding than ^111^In-oxine. Intracellular protein binding of ^89^Zr accounts for the ability of ^89^Zr-oxine labeling to successfully track cells in vivo long-term on PET.

## 1. Introduction

Cell-based therapies, including those using tumor-infiltrating T cells, chimeric antigen receptor (CAR)-T cells, and stem cells, have become major therapeutic strategies for cancers and other diseases [1,2,3]. Cell-based therapies rely on the transferred cells arriving at the target organ or tissue in sufficient numbers to have a therapeutic effect, but this is difficult to non-invasively ascertain. Hence, to optimize the therapeutic potential of cell-based therapies, the ability to measure the distribution of therapeutic cells throughout the body and assess the effects of cell engineering on migration, targeting and engraftment is crucial [4,5]. Zirconium-89 (^89^Zr)-oxine has been developed as an ex vivo labeling agent for tracking cells in vivo over multiple days using positron emission tomography (PET) imaging [6,7]. Unlike indium-111 (^111^In)-oxine or technetium-99m (^99m^Tc)-hexamethylpropyleneamine oxime (HMPAO) cell labeling that utilizes single-photon emission computed tomography (SPECT) imaging [8,9,10,11], the utilization of PET enables cell detection with higher sensitivity, lower radioactivity, and, thus, lower radiotoxicity to the cells. Furthermore, the short half-life of ^99m^Tc (6-h) is a limitation because it greatly shortens the imaging window for tracking cells [12,13]. In comparison, ^89^Zr and ^111^In have longer half-lives of 3.3 days and 2.7 days, respectively, making them more suitable for the longer-term tracking of cells [5,6,7,8].

^89^Zr-oxine ex vivo cell labeling has been applied to the tracking of many different cell types, such as T cells, natural killer (NK) cells, dendritic cells (DCs), hematopoietic stem cells, eosinophils and plasma cells, in preclinical mouse and non-human primate studies, and has proven to be safe, exhibiting negligible radiotoxicity, when optimal labeling doses are used [6,14,15,16,17,18,19]. Studies using labeled CAR-T cells indicated no difference in the cytokine production and cytotoxic function between the labeled and non-labeled cells [20,21]. Clinical application of ^89^Zr-oxine cell labeling and tracking has recently started [22].

^89^Zr-oxine and ^111^ln-oxine share structural similarities and labeling protocols. Previous reports indicated that post-labeling dissociation of the ^111^ln-oxine complexes within the cell leads to the intracellular trapping of the ^111^ln while releasing the oxine [9,23,24,25]. Similar studies for ^89^Zr-oxine have not been performed. Despite their differences in oxidation state, +3 for indium (ln) and +4 for zirconium (Zr), both metals have shown similarities in reactivity and preferred ligand type [26]. Thus, we postulated that both isotopes share similar cellular binding and intracellular distribution.

In this study, we aimed to determine the intracellular distribution and stability of protein binding of ^89^Zr-oxine, compared to ^111^ln-oxine, after cell labeling.

## 2. Materials and Methods

### 2.1. Animals

C57BL/6 mice were purchased from Jackson Laboratories and handled in accordance with a protocol approved by the National Cancer Institute Animal Care and Use Committee. Either male or female mice aged 8–12 weeks of age were used for naïve T cell purification, and those aged 10–24 weeks were used for other experiments.

### 2.2. Cell Culture

RPMI 1640 medium (Thermo Fisher Scientific, Waltham, MA, USA) supplemented with 2 mmol/L L-glutamine (Thermo Fisher Scientific), 100 IU/mL penicillin/100 µg/mL streptomycin (Thermo Fisher Scientific), 10% fetal calf serum (FCS, Gemini Bio Products, West Sacramento, CA, USA), and 0.05 mmol/L 2-mercaptoethanol (Millipore Sigma, Burlington, MA, USA) was used for all cell cultures. To generate bone marrow-derived monocytes and macrophages, bone marrow cells flushed out of the femurs and tibias of mice were grown for 5 days (for monocytes) and 9 days (for macrophages) with the addition of 20 ng/mL and 10 ng/mL, respectively, of macrophage-colony stimulating factor (M-CSF, Peprotech, Cranbury, NJ, USA). For differentiation of dendritic cells (DCs), bone marrow cells were grown in 20 ng/mL of granulocyte-macrophage-colony stimulating factor (GM-CSF, Peprotech) for 9 days. Naïve T cells were purified from the spleen of mice as follows: The spleen was passed through a 70 µm-pore strainer (Corning Life Sciences, Tewksbury, MA, USA) in phosphate-buffered saline (PBS), and the obtained single cell suspension was layered over Cell Separation Medium (Lonza, Walkersville, MA, USA), followed by centrifugation at 240× *g* for 12 min with low acceleration/deceleration. The cells in the interphase were collected, washed with PBS containing 0.5% bovine serum albumin (Miltenyi, Gaithersburg, MA, USA) and 2 mmol/L ethylenediaminetetraacetic acid (EDTA), and underwent a positive selection of T cells using CD8 and CD4 T cell magnetic beads (Miltenyi) and an LS column (Miltenyi) according to the manufacturer’s instructions. EL4 (murine lymphoma) cells and DC2.4 cells (murine DC line) were purchased from American Type Culture Collection and Millipore Sigma, respectively. Kit225K6 (human T cell lymphoma) cells were kindly provided by the late Dr. Thomas Waldmann, National Cancer Institute, National Institutes of Health. MC38 (murine colon adenocarcinoma) cells were purchased from Kerafast (Boston, MA, USA).

Live cells were counted using a LUNA-FL Dual Fluorescence Cell Counter and Acridine Orange/Propidium Iodide Stain, following the manufacturer’s instructions (Logos Biosystems, Anyang, Republic of Korea), throughout the study.

### 2.3. Flow Cytometry Analysis

Purified naïve T cells were stained with anti-CD8 (clone 53.67) and CD4 (clone RM4-5) antibodies conjugated with fluorescein isothiocyanate (FITC) and allophycocyanin (APC), respectively (Thermo Fisher Scientific), and run on a flow cytometer [FACSCaliber, Becton Dickinson Biosciences (BD), Franklin Lakes, NJ, USA] to examine their purity. When determining forward scatter (FSC), which reflects cellular size, and side scatter (SSC), which reflects granular/vesicular content, of various cell types, a 300 µL cell suspension in PBS with 0.1% FCS (FCM buffer) was prepared, and 0.3 µL of 10 mg/mL propidium iodide (Sigma Aldrich, St. Louis, MO, USA) was added to stain dead cells immediately before the acquisition of the data. The acquisition settings, including voltage and gain, were kept consistent across the cell types, allowing for comparison of the FSC and SSC profiles of live (propidium iodide negative) cells. All flow cytometry data were analyzed using the FlowJo software ver.10 (BD), including the calculation of mean FSC and mean SSC values for each sample, representing cell size and granular/vesicular content, respectively. The determined FSC and SSC values of the samples were further averaged across the replicates in each cell type, and standard deviations were calculated.

### 2.4. Total Protein Measurement of Cell Lysates

Two million cells were lysed with 200 µL of M-PER Mammalian Protein Extraction Reagent (Thermo Fisher Scientific), vortexed, and incubated on a shaker for 10 min. The samples were centrifuged at 6760× *g* for 5 min and the supernatants were collected. The sample was then subjected to a DC Protein Assay Kit (Bio-Rad, Hercules, CA, USA) to measure the total protein concentration using a spectrophotometer at 750 nm absorbance.

### 2.5. ^89^Zr-Oxine Production and Cell Labeling

^89^Zr-chloride was produced from ^89^Zr-oxalate generated at the cyclotron facility of the Clinical Center, National Institutes of Health, and was used to synthesize the ^89^Zr-oxine complex following a previously established protocol [6,27]. Briefly, 2 µL Tween 80 (20%), oxine (102 µL of 20 mmol/L in 0.04 N HCl), and ^89^Zr-chloride (60 µL, 25.9–40.5 MBq) were mixed and vortexed in a 1.5 mL tube, followed by neutralization using NaHCO_3_ (500 mmol/L). The final solution pH ranged between 7 and 7.3.

To determine the ^89^Zr incorporation levels and labeling efficiencies in various cell types, cells in PBS were incubated with 37 kBq of ^89^Zr-oxine solution per 10^6^ cells at a 30:1 volume ratio for 15 min. In select experiments, the incubation was shortened to 5 min to evaluate the kinetics of protein binding of ^89^Zr. After the incubation, 500 µL culture medium was added to the incubation mixture, centrifuged at 1900× *g* for 2 min, and the supernatant was removed. The cells were washed twice with 800 µL culture medium, resuspended in 800 µL PBS, transferred to a new tube, and centrifuged again. ^89^Zr activity was measured during the incubation and after washing using a micro-dose calibrator built in-house [28], and the activity incorporation yield (labeling efficiency) was calculated. To determine the ^89^Zr incorporation thresholds, 2 × 10^6^ EL4, DC2.4, or Kit225K6 cells were incubated with increasing doses of ^89^Zr-oxine, aiming to label the cells at 18.5, 37, 74, 148, 296, and 592 kBq per 10^6^ cells, based on the labeling efficiencies obtained above.

### 2.6. ^111^In-Oxine Cell Labeling

^111^In-oxine was purchased from GE Healthcare (Chicago, IL, USA, specific activity 59.2 kBq/mL). To determine the ^111^In incorporation levels, 2 × 10^6^ EL4 and DC2.4 cells were incubated with 370 kBq per 10^6^ cells in 100 µL PBS for 15 min at room temperature. After incubation, the samples underwent the same washing procedures as ^89^Zr-oxine labeled cells. The incubation and incorporated activities were measured using a dose calibrator (Capintec, Florham, NJ, USA).

### 2.7. Trichloroacetic Acid Protein Precipitation

To determine the intracellular protein binding of ^89^Zr-oxine or ^111^In-oxine, 2 × 10^6^ labeled EL4, Kit225K6, or DC2.4 cells were lysed with 200 µL of M-PER Mammalian Protein Extraction Reagent (Thermo Fisher Scientific) immediately after labeling. PBS containing either ^89^Zr-oxine alone or ^111^In-oxine alone was used as a protein-free control in the relevant assays. One mL of 10% ice-cold trichloroacetic acid (TCA) in PBS was added to each lysate, incubated on ice for 10 min, and then centrifuged at 18,400× *g* for 5 min. The supernatants were separated from the pellets, and the activity in the supernatants (non-bound) and pellets (protein-bound) was measured using a γ-counter (Wizard 2, Perkin-Elmer, Shelton, CT, USA).

### 2.8. Cellular Fractionation and Western Blotting

Five million EL4, DC2.4, and Kit225K6 cells labeled with ^89^Zr-oxine (185–259 kBq/10^6^ cells) underwent subcellular fractionation using a Subcellular Protein Fractionation Kit for Cultured Cells (Thermo Fisher Scientific), which separates the fractions with approximately 90% accuracy, according to the manufacturer’s instructions. Briefly, the cell pellet volume was estimated to be approximately 30 µL, and the cells were fractionated into cytoplasmic, membrane-bound, soluble nuclear, chromatin-bound, and cytoskeletal fractions in 300 µL Cytoplasmic Extraction Buffer, 300 µL Membrane Extraction Buffer, 150 µL Nuclear Extraction Buffer (NEB), 150 µL Chromatin-bound Extraction Buffer (NEB with 7.5 µL of 100 mM CaCl2 and 4.5 µL of Micrococcal Nuclease), and 150 µL Pellet Extraction Buffer, each containing protease inhibitors, respectively. The ^89^Zr activity in each cellular component was measured using a γ-counter (Perkin-Elmer). The quality of the fractionation was determined by western blot analysis by running 30 µL each of cytoplasmic and membrane-bound lysates and 15 µL each of soluble nuclear, chromatin-bound, and cytoskeletal lysates (i.e., 1/10th of each fractionated samples) mixed with 10 µL and 5 µL of 4× Laemmli Sample Buffer (Bio-Rad), respectively, on either 4–20% or 10% tris-glycine gels (Thermo Fisher Scientific). The electrophoresed samples were blotted against PVDF membranes (Immobilon-P, Millipore Sigma). A set of membranes was set aside for autoradiography. The PVDF membranes were blocked for non-specific binding with a PBS Blocking Buffer (Thermo Fisher Scientific) and probed with antibodies against histone H3 (clone 3H1), lysine-specific demethylase 1 (LSD1, clone C69G12), or vimentin (clone D21H3) purchased from Cell Signaling Technology for overnight at 4 °C, or with anti-Mek-1 (clone H-8, Santa Cruz Biotechnology, Dallas, TX, USA) or anti-calnexin (polyclonal, Enzo, Farmingdale, NY, USA) antibody for 1 h at room temperature. Then the membranes were washed using PBS with 0.1% Tween 20 for 1 h and incubated with a horseradish peroxidase-conjugated anti-rabbit antibody (1:20,000 dilution, Cytiva/GE Healthcare, Marlborough, MA, USA) or anti-mouse IgG (1:1000 dilution, Pierce, Appleton, WI, USA). The blots were developed using SuperSigna West Dura Extended Duration Substrate (Thermo Fisher Scientific), following the manufacturer’s instructions, and analyzed using a Chemi Doc imaging system (Bio-Rad). ^111^In-oxine labeled EL4 and DC2.4 cells (139 and 141 kBq/10^6^ cells, respectively) were fractionated into subcellular compartments in a similar manner.

### 2.9. Autoradiography

PVDF membranes obtained from Western blotting of subcellular fractionated samples of the cells labeled with ^89^Zr-oxine or ^111^In-oxine were subjected to autoradiography. Phosphor screens (BAS-IP SR 2025, Cytiva/GE Healthcare) were exposed to the PVDF membranes for 7 days in cassettes and analyzed on a phosphor imager (Typhoon FLA 9000 Gel Scanner, Cytiva/GE Healthcare) at a 0.25 µm resolution.

### 2.10. Analysis of ^89^Zr Retention in Labeled Cells

Kit225K6 cells (5.6 × 10^6^ cells) were labeled with increasing doses of ^89^Zr-oxine. Labeled cells were plated at 3.5 × 10^5^ cells per 350 µL in triplicates. At each time point indicated, cell number and viability were examined using a LUNA-FL Dual Fluorescence Cell Counter and Acridine Orange/Propidium Iodide Stain, and cell-associated activity was measured with a γ-counter after centrifugation (1900× *g* for 2 min) and removal of the supernatants. The radioactivity results were decay corrected.

### 2.11. Biodistribution of ^89^Zr-Labeled and ^111^In-Oxine-Labeled Cells

Six million DCs (differentiated from the bone marrow collected from female mice) labeled with either ^89^Zr-oxine (29.6 kBq/10^6^ cells) or ^111^In-oxine (76.0 kBq/10^6^ cells) were intravenously administered to female recipient mice. One day and 7 days later, the mice were euthanized by CO_2_ inhalation, weighed, had blood withdrawn, and indicated organs and tissues were harvested. A femur was collected from each mouse as a bone/bone marrow sample. The collected blood and organs/tissues were weighed, and their radioactivity was measured using a γ-counter. The percent injected dose per gram of tissue (%ID/g), normalized to a 20-g mouse, was calculated using the following formula: %ID/g = [decay-corrected radioactivity of the tissue (cpm)]/[injected activity (cpm)]/[tissue weight (g)] × 100 × [body weight (g)]/20 (g), where cpm stands for count per minute.

### 2.12. Statistical Analysis

The statistical analysis was conducted using GraphPad Prism software (ver. 10.3.1, GraphPad Software). Correlation analyses were based on the Pearson r value. A two-tailed unpaired Student’s *t*-test was utilized to compare 1 variable between two groups. For the comparison of multiple groups involving 1 variable, an ordinary one-way analysis of variance (ANOVA) was employed, and for that involving 2 variables, a two-way ANOVA was used. For analyzing 2 variables over time, a repeated measure two-way ANOVA was used. Statistical significance was defined as *p*-values less than 0.05, where ‘n’ represents the number of replicates.

## 3. Results

### 3.1. Cellular Incorporation of ^89^Zr-Oxine Positively Correlates with Cellular Protein Mass

**Figure 1:**
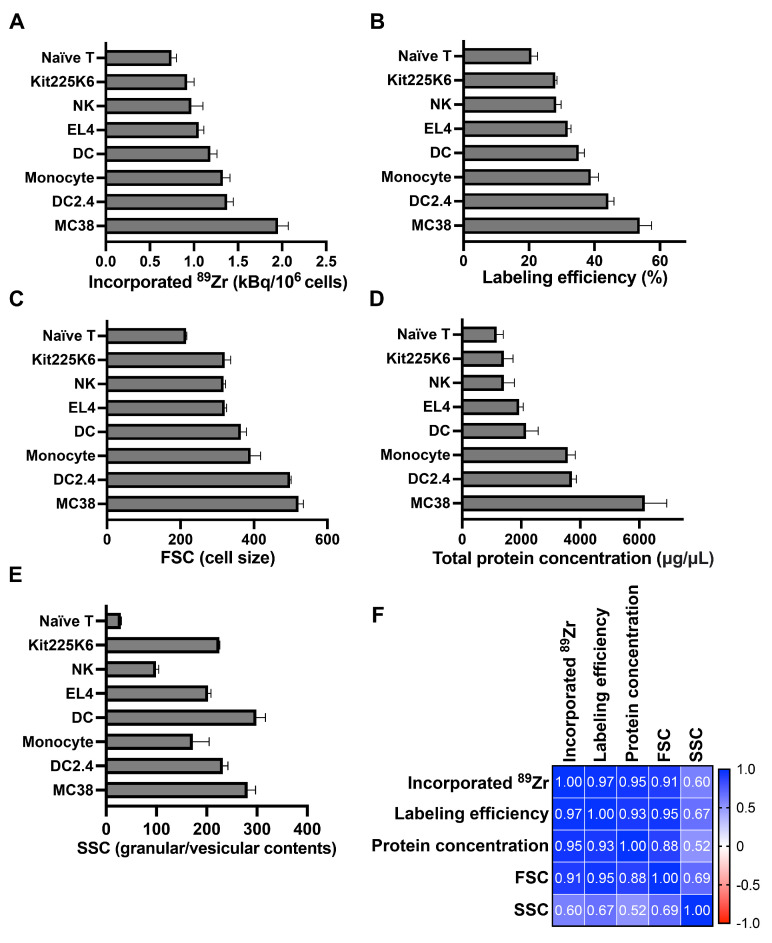
^89^Zr-oxine cell labeling positively correlates with the protein mass and cell size. (**A**) Various cell types incubated with 37 kBq/10^6^ cells of ^89^Zr-oxine incorporated different amounts of activity (n = 3–6). (**B**) Labeling efficiency (percentage of incorporated activity vs. incubation activity) was calculated for each cell type (n = 3–6). (**C**) Flow cytometry was performed to determine the forward scatter (FSC) values that reflect cellular size (n = 3–6). (**D**) Various cell types were lysed at 100 µL/10^6^ cells, and total protein concentration of each lysate was measured as a readout for protein mass of a cell (n = 3–6). (**E**) Using flow cytometry, the side scatter (SSC) value reflecting vesicular contents was obtained for each cell type (n = 3–6). All data in (**A**–**E**) are represented as mean ± standard deviation. (**F**) Correlation analysis using the values shown in (**A**–**E**) indicated strong positive correlation between ^89^Zr activity incorporation or labeling efficiency with protein concentration and cell size (FSC). Pearson r values are shown.

We have previously demonstrated that ^89^Zr-oxine cell labeling occurs in a passive manner via cell permeabilization without relying on cellular bioactivity [6] and can label various cell types [6,14,15,16]. To further determine the parameters that affect cellular incorporation of ^89^Zr-oxine, we subjected naïve T cells, NK cells, DCs and monocytes, as well as EL4, DC2.4, MC38, and Kit225K6 cells to labeling with ^89^Zr-oxine. The purity of naïve T cells was greater than 97.5%, as determined by flow cytometry analysis. Incubation with 37 kBq/10^6^ cells of ^89^Zr-oxine resulted in the activity uptake per 10^6^ cells from a low of 0.75 ± 0.06 kBq (naïve CTLs) to a high of 1.95 ± 0.12 kBq (MC38 cells) (Figure 1A and Table S1), with the labeling efficiencies ranging from 20.76 ± 1.82% (naïve CTLs) to 53.84 ± 3.65% (MC38) (Figure 1B and Table S1). Based on the FSC analysis of the cells by flow cytometry (Figure 1C and Table S1), cell size had a positive correlation with the ^89^Zr-oxine incorporation and thus the labeling efficiency. We next asked if the protein mass of a cell determines the amount of ^89^Zr incorporation. We generated a whole cell lysate in a pre-determined volume and measured the total protein concentration as a readout of the protein mass. The cells with a greater total protein concentration showed higher ^89^Zr-oxine incorporation (Figure 1D and Table S1). Of note, the granular/vesicular contents of the cells represented by the SSC of flow cytometry analyses did not show the same trend by cell type (Figure 1E and Table S1). Statistical correlation analysis demonstrated a high Pearson correlation coefficient of 0.95 between the total protein concentration (protein mass) and incorporated ^89^Zr activity (Figure 1F). The correlation coefficient between the FSC and incorporated ^89^Zr activity was also high (0.91), indicating that cellular uptake of ^89^Zr-oxine can be estimated by the cellular size.

### 3.2. ^89^Zr Binds to Intracellular Proteins

**Figure 2:**
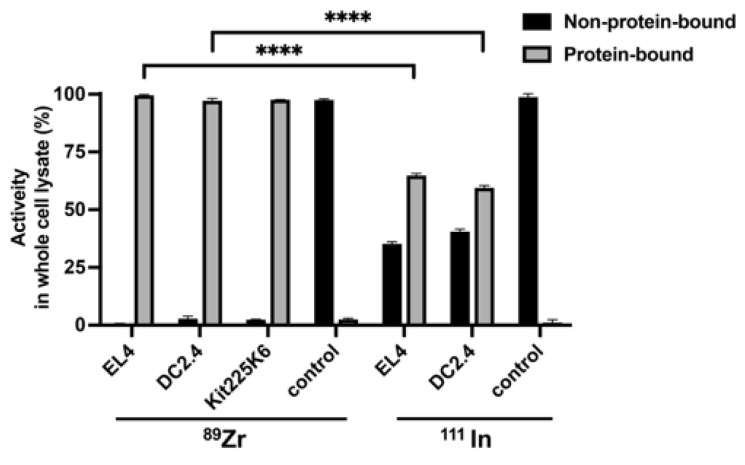
^89^Zr incorporated into cells is protein-bound. Trichloroacetic acid (TCA) protein precipitation of lysates generated from 2 × 10^6 89^Zr-oxine labeled EL4, DC2.4, and Kit225K6 cells demonstrated that virtually all activity was protein-bound (n = 3). By contrast, more than one-third of the activity was non-protein-bound in the cells labeled with ^111^In-oxine (n = 4), demonstrating significant differences in % protein binding with ^89^Zr-oxine labeling. PBS containing either ^89^Zr-oxine alone or ^111^In-oxine alone was used as a control in the relevant assays, and the addition of TCA to these control samples did not precipitate the activity. Data are represented as mean ± standard deviation. ****: *p* < 0.0001 by two-way ANOVA. Radioactivity of the protein-bound fraction in each cell lysate was significantly higher than that in the relevant control (*p* < 0.0001, not indicated in the graph).

Next, we investigated the fate of ^89^Zr after cell labeling, specifically if ^89^Zr binds to cellular proteins. ^89^Zr-oxine labeled cells were lysed, and TCA precipitation of proteins was performed to separate protein-bound and non-protein-bound ^89^Zr. The results indicate that virtually all ^89^Zr was protein-bound in all cell types tested; 99.5 ± 0.3% in EL4 cells, 97.1 ± 1.1% in DC2.4 cells, and 97.6 ± 0.1% in Kit225K6 cells (Figure 2). In the control without proteins (^89^Zr-oxine in PBS), 97.5 ± 0.5% of ^89^Zr was found as non-precipitated, non-protein-bound activity in the solution. For comparison, we performed similar experiments using ^111^In-oxine labeled EL4 and DC2.4 cells. Although ^111^In was also protein-bound, the fraction was significantly lower than that of ^89^Zr; 64.8 ± 0.9% of ^111^In in EL4 and 59.4 ± 1.0% in DC2.4 cells were protein-bound (Figure 2).

### 3.3. ^89^Zr Localizes in the Cytoplasm, Membranes, and the Nucleus, Whereas ^111^In Primarily Locates in the Cytoplasm

**Figure 3:**
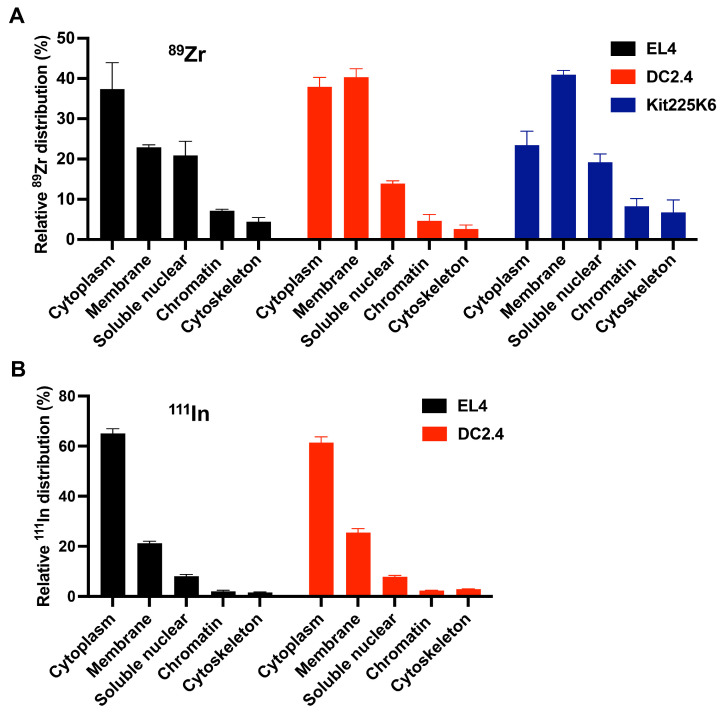
^89^Zr-oxine cell labeling leads to distinct intracellular distribution of ^89^Zr by cell type, predominantly in the cytoplasm, membranes, and nucleus, whereas ^111^In-oxine labeling results in the activity limited to the cytoplasm. (**A**) ^89^Zr-oxine labeled EL4, DC2.4, and Kit225K6 cells were fractionated into the subcellular compartments indicated, and ^89^Zr activity in each fraction was measured. ^89^Zr primarily localized in the cytoplasm, membrane, and soluble nuclear fractions, showing different distribution patterns among the cell types (n = 4 for EL4 and DC2.4 cells, n = 3 for Kit225K6 cells). (**B**) Subcellular fractionation of ^111^In-oxine labeled EL4 and DC2.4 cells was performed, followed by activity measurement for each fraction. ^111^In primarily localized in the cytoplasm, membrane, and soluble nuclear fractions, showing different distribution patterns among the cell types (n = 3). All data are represented as mean ± standard deviation.

To determine the intracellular location of the ^89^Zr and ^111^In, cells labeled with ^89^Zr-oxine and ^111^In-oxine were fractionated into their core cellular components. The ^89^Zr activity measurements of the fractionated components revealed that most of the radioactivity was detected in the cytoplasmic, membrane, and soluble nuclear fractions in EL4 (81.2 ± 10.3%), DC2.4 (92.2 ± 3.0%), and Kit225K6 (83.6 ± 5.5%) cells, with different distribution patterns among these fractions by cell type (Figure 3A). Of note, the membrane fraction contains both the cytoplasmic and intracellular membrane components. By contrast, the activity distribution of ^111^In-oxine labeled cells showed 65.1 ± 1.8% and 61.5 ± 2.2% of ^111^In activity localizing in the membrane in EL4 and DC2.4 cells, followed by 21.2 ± 0.8% and 25.5 ± 1.6% in the cytoplasm, respectively (Figure 3B). Unlike ^89^Zr, intracellular distribution of ^111^In did not differ by the cell types examined, and the activity in the soluble nuclear fraction was low in all cases. Of note, western blotting of each representative fraction marker confirmed the quality of the fractionated samples (Figure S1).

### 3.4. ^89^Zr-Oxine Labels Multiple Cellular Proteins of Different Sizes

**Figure 4:**
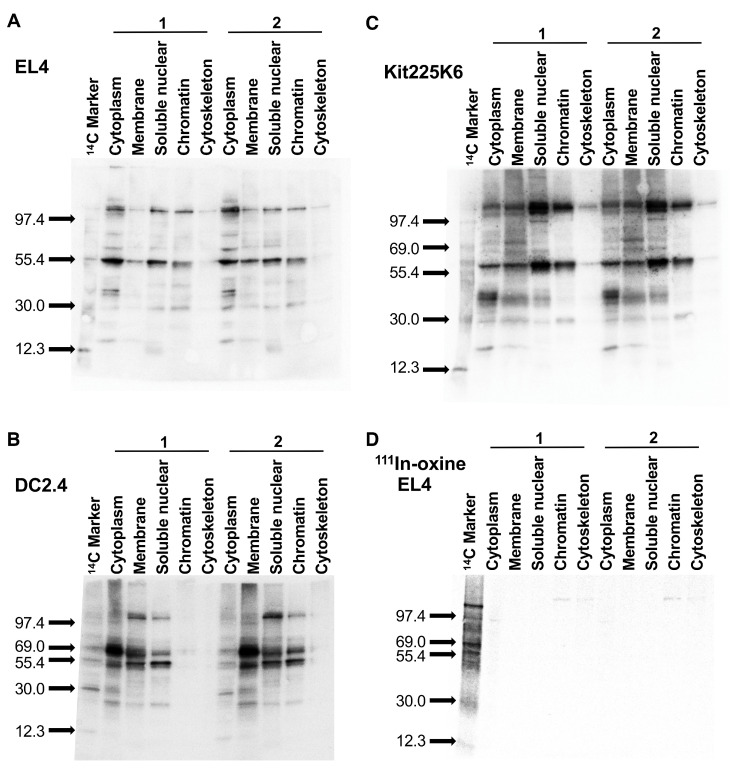
^89^Zr-oxine labels multiple cellular proteins of different sizes in various subcellular compartments. (**A**–**C**) Autoradiography of the membranes obtained from western blotting of subcellular fractions of ^89^Zr-oxine labeled EL4 (**A**), DC2.4 (**B**), and Kit225K6 (**C**) cells. Multiple bands in each fraction indicate the binding of ^89^Zr to multiple proteins of different sizes. The pattern of protein binding of ^89^Zr differed by cell type. Representative results of more than three independent experiments showing duplicated sample preparations for each cell type. (**D**) Autoradiography of ^111^In-oxine labeled EL4 cell subcellular fractions only showed extremely faint bands in the cytoplasmic, chromatin-bound, and cytoskeletal fractions. Representative results of two independent experiments, showing duplicated sample preparations.

We next asked if ^89^Zr binds to a specific protein after ^89^Zr-oxine labeling. Autoradiography of subcellular fractionations of EL4, DC2.4, and Kit225K6 cells demonstrated multiple ^89^Zr-bound proteins that differ by the fraction and cell type. The results indicate that ^89^Zr was bound to diverse intracellular proteins of different sizes in different subcellular components (Figure 4A–C). By contrast, the autoradiography of ^111^In-oxine labeled EL4 cell fractions showed no bands except for an extremely weak band shown in the cytoplasmic, nuclear, and cytoskeletal fractions. The results from ^111^In-oxine labeled cell fractions indicate that ^111^In did not strongly bind to proteins.

### 3.5. Intracellular Protein Binding of ^89^Zr Occurrs Rapidly

**Figure 5:**
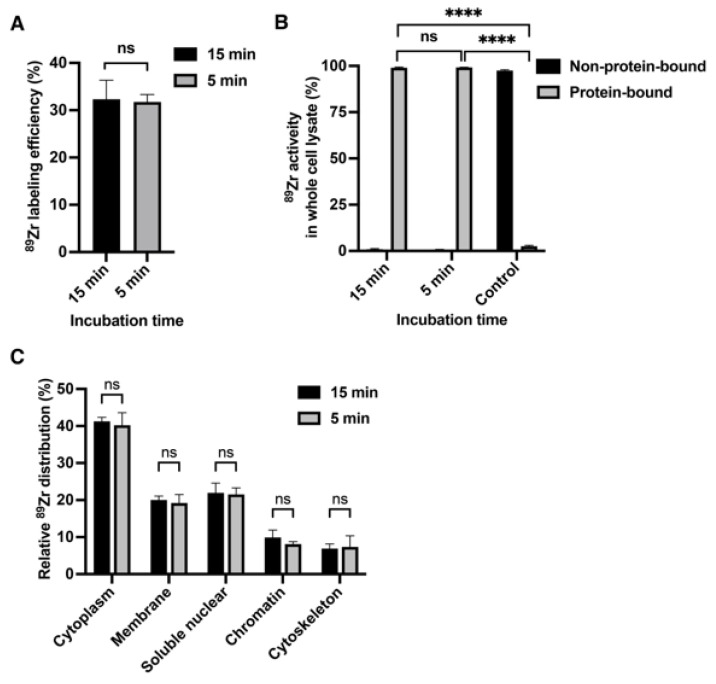
Intracellular protein binding of ^89^Zr-oxine occurs rapidly. (**A**) EL4 cells incubated with ^89^Zr-oxine for 15 min and 5 min showed similar labeling efficiency with no statistical difference (ns: not significant by Student’s two-tailed *t*-test, n = 3). (**B**) EL4 cells incubated with ^89^Zr-oxine for 5 min showed virtually all activity bound to protein(s) similar to the cells incubated for 15 min, as indicated by the TCA protein precipitation of whole cell lysates. PBS containing ^89^Zr-oxine alone was used as a control, which did not precipitate the activity (n = 3, ns: not significant, ****: *p* < 0.0001, by one-way ANOVA). (**C**) Five min incubation was sufficient to similarly label intracellular proteins as 15 min incubation in EL4 cells (n = 3, ns: not significant, by two-way ANOVA). All data are represented as mean ± standard deviation.

We then evaluated the effects of shortening the incubation time of ^89^Zr-oxine cell labeling on intracellular distribution using EL4 cells. Under incubation times of 15 min vs. 5 min, we noted no significant difference in labeling efficiency, percent of protein-bound activity by TCA precipitation, or intracellular distribution. In both experimental conditions, the labeling efficiencies were 32.3 ± 4.0% (15 min) and 31.8 ± 1.5% (5 min), with >99% of the radioactivity protein-bound (Figure 5A,B). The intracellular distributions of ^89^Zr activity were also similar between the two labeling conditions (Figure 5C). These results indicate that ^89^Zr-oxine labeling of cells and subsequent binding of ^89^Zr to subcellular proteins occurs rapidly.

### 3.6. ^89^Zr-Oxine Incorporation Saturation Depends on Cell Type

**Figure 6:**
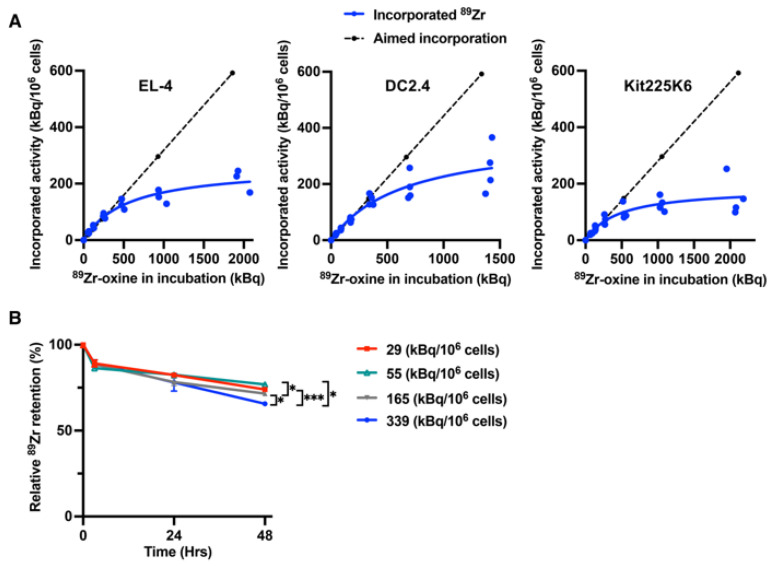
^89^Zr-oxine incorporation demonstrates a cell type-dependent saturation, and an activity lower than the saturation shows better retention. (**A**) Saturation assay shows the maximum of ^89^Zr-oxine incorporation in EL4, DC2.4, and Kit225K6 cells that were incubated with increasing doses of ^89^Zr-oxine calculated from the labeling efficiency shown in Figure 1B for the following targeted incorporation doses: 18.5 KBq/10^6^, 37KBq/10^6^, 74 kBq/10^6^, 148 kBq/10^6^, 296 kBq/10^6^, and 592 kBq/10^6^ cells (broken line). Incorporated activity plateaued at higher doses, indicating the presences of saturation thresholds. EL4 and DC2.4 showed a threshold point around 90 kBq/10^6^ cells, while that of Kit225K6 was around 60 kBq/10^6^ cells (n = 4 for EL4 and Kit225K6 cells, n = 3 for DC2.4 cells). (**B**) Kit225K6 cells labeled with ^89^Zr-oxine at 29, 55, 165, and 339 kBq/10^6^ cells were cultured, and decay-corrected cell-associated activity was examined over 48 h (also see Figure S2A). Relative ^89^Zr retention compared to the activity immediately after the labeling (0 h) is plotted for each labeling dose (n = 3, *: *p* < 0.05, ***: *p* < 0.001, by repeated measure two-way ANOVA). Data are represented as mean ± standard deviation.

Having established that different cell types have different labeling efficiencies, which are determined by cell size and protein mass, we determined if there was an activity incorporation threshold to ^89^Zr-oxine labeling. Using EL4, DC2.4, and Kit225K6 cells, we compared expected and actual ^89^Zr activity uptake. We labeled the cells aiming to achieve increasing incorporation activities based on the labeling efficiency of the cell type (Table S1); we calculated the ^89^Zr-oxine incubation activity required to achieve the aimed-for incorporation activity. However, we observed plateauing of the incorporated activity at high incubation doses, indicating that a labeling threshold existed (Figure 6A). The approximate threshold points at which calculated activity incorporation began to diverge from the actual incorporation were approximately 90 kBq/10^6^ cells for EL4 and DC2.4 cells and 60 kBq/10^6^ cells for Kit225K6 cells, which corresponded to approximately 1/3 of the maximum incorporation activity, estimated from the fit curves, for EL4 and Kit225K6 cells, and approximately 1/4 for DC2.4 cells.

### 3.7. ^89^Zr Retention Is Affected by the Incorporation Threshold

The presence of a labeling saturation in ^89^Zr-oxine cell labeling suggests that ^89^Zr-oxine labeling above the threshold point might be unstable due to a shortage of intracellular proteins. Unstable ^89^Zr could more easily be released from the cells than ^89^Zr labeled at a sub-saturating concentration. To test this hypothesis, we labeled Kit225K6 cells, which have relatively low protein mass (Figure 1D), at varying specific activities (kBq/10^6^ cells) and examined the decay-corrected activity retention over a 48 h period (Figure 6B and Figure S2A). The 48 h observation period was selected to minimize the death of the cytokine-dependent Kit225K6 cells that would affect ^89^Zr retention. The cell viability and number did not differ among the cells labeled at different specific activities (Figure S2B). Approximately 80% of decay-corrected ^89^Zr activity was retained in the first 24 h, after which the cells labeled well above the saturation threshold point of 60 kBq/10^6^ cells showed significantly less ^89^Zr retention than the cells labeled below the saturation point.

### 3.8. ^89^Zr-Oxine Labeled Cells Retain the Activity Better than ^111^In-Oxine Labeled Cells In Vivo

Our in vitro data (Figure 2 and Figure 4) demonstrated higher protein binding and labeling stability with ^89^Zr-oxine compared to ^111^In-oxine. To this end, we assessed in vivo retention of radioactivity by infusing mice with DCs labeled with either ^89^Zr-oxine or ^111^In-oxine. Since minimizing radioactivity release due to cell death is critical in this context, we used DCs, which are known to be relatively radioresistant. The biodistribution of the labeled DCs was examined one day after intravenous infusion, when their distribution reaches a steady state [6] and viability remains high, and again on day 7. Because higher activity is required for SPECT imaging compared to PET, DCs were labeled with ^111^In-oxine at a higher dose of 76.0 kBq/10^6^ cells compared to the ^89^Zr-oxine dose of 29.6 kBq/10^6^ cells, while keeping the infused cell number of constant at 6 × 10^6^ cells. The infusion activity was 456 kBq for ^111^In-oxine labeled DCs and 177.6 kBq for ^89^Zr-oxine labeled DCs.

**Figure 7:**
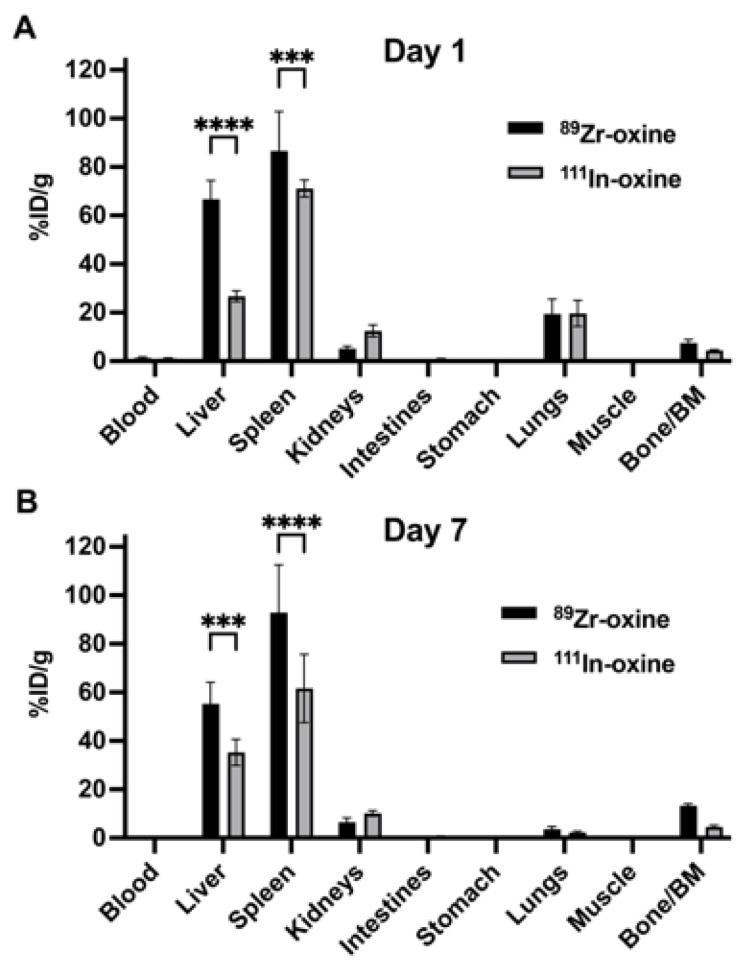
Biodistribution of ^89^Zr-oxine labeled cells showed higher activity retention compared to ^111^In-oxine labeled cells. (**A**,**B**) The graphs show %ID/g values of intravenously infused DCs (6 × 10^6^ cells) labeled with ^89^Zr-oxine (29.6 kBq/10^6^ cells) or ^111^In-oxine (76.0 kBq/10^6^ cells) in mice 1 day (**A**) and 7 days (**B**) after infusion. The cells primarily migrated to the liver and spleen, regardless of the labeling method. However, the %ID/g values of ^89^Zr were significantly higher than those of ^111^In in both organs, indicating greater activity retention in ^89^Zr-oxine labeled cells (n = 4, ***: *p* < 0.001, ****: *p* < 0.0001, by two-way ANOVA). Data are decay corrected, normalized to a 20-g mouse, and represented as mean ± standard deviation. BM: bone marrow.

Biodistribution analysis of ^89^Zr and ^111^In activity revealed that infused DCs, regardless of the radiolabel used, primarily migrated to the liver and spleen (Figure 7). However, ^89^Zr showed significantly higher %ID/g values than ^111^In in both organs on days 1 and 7, suggesting greater retention of activity in ^89^Zr-oxine labeled cells than ^111^In-oxine labeled cells. Slightly higher ^111^In activity than ^89^Zr was observed in the kidneys, while ^89^Zr activity was higher than ^111^In in the bone/bone marrow, though these differences were not statistically significant. These findings are consistent with renal excretion of released ^111^In and bone uptake of released ^89^Zr, respectively.

## 4. Discussion

Cell-based therapies using T cells or NK cells depend on the successful migration of the cells to a target organ or tissue. However, such cell tracking is difficult to perform non-invasively. ^89^Zr-oxine has emerged as the leading contender for ex vivo labeling of therapeutic cells to enable visualization and quantification of their distribution and migration kinetics in the body after infusion [6,7,15,18]. In this study, we examined how ^89^Zr-oxine distributes within cells and what influences its cellular retention to enable successful imaging by PET.

Our previous studies demonstrated that ^89^Zr-oxine can label many different cell types, albeit at different efficiencies [6]. In this study, we demonstrated that the percent of ^89^Zr uptake (labeling efficiency) and activity incorporated positively correlated with the protein mass and size of the cell (r > 0.91). ^89^Zr-oxine demonstrated high cellular protein binding (>97%), accounting for the dependence on the protein mass of cells. Once labeled with ^89^Zr-oxine, breakdown of the ^89^Zr-oxine complex followed by binding of ^89^Zr to cellular proteins occurred relatively rapidly, and trapped the ^89^Zr radiometal inside the cells causing little (<3%) free ^89^Zr. Shortening the incubation time from 15 min to 5 min did not change the intracellular protein binding and distribution of ^89^Zr. This observation is supported by the known kinetics of neutral and lipid-soluble complexes [29], of which the ^89^Zr-oxine complex is an example. The differences in the granular/vesicular contents of each cell type did not affect the uptake and retention of ^89^Zr-oxine, as the labeling relies on the membrane-permeable nature of the ^89^Zr-oxine complex [6,8].

The protein binding and intracellular distribution of ^89^Zr were compared to those of ^111^In-oxine labeling. In contrast to ^89^Zr-oxine, ^111^In-oxine labeled cells showed only 59–65% of activity binding to proteins, suggesting possible instability of the ^111^In complex within the cell. Of note, we used higher doses of ^111^In-oxine to label cells compared to ^89^Zr-oxine, because of the requirement of higher radioactive doses for imaging ^111^In by SPECT [30]. If labeling saturation exists with ^111^In-oxine, similar to ^89^Zr, the high dose used for labeling might have lowered the percentage of intracellular protein-bound ^111^In. However, we did not observe meaningful ^111^In-bound proteins on autoradiography, indicating more unstable protein binding for ^111^In than ^89^Zr. Nevertheless. it was surprising that so many ^89^Zr-protein complexes survived the western blotting procedure and were detected by autoradiography in our study.

In ^89^Zr-oxine labeled cells, the majority of ^89^Zr was found in the cytoplasmic fraction, cytoplasmic and intracellular membrane fraction, and soluble nuclear fraction, but the distribution ratios differed among EL4, DC2.4, and Kit225K6 cells. We attribute this to differences in protein concentrations across various cell components among different cell types. EL4 cells have a relatively smooth surface and are round, as compared to DC2.4 cells, which are long and branching. DC2.4 thus provides a greater surface area for the membrane, explaining higher membrane protein labeling with ^89^Zr. Kit225K6 cells are small, irregular, and contain a significant number of vesicles, which helps explain the high binding of ^89^Zr to the proteins in the membrane. In contrast to ^89^Zr, the distribution of ^111^In after ^111^In-oxine cell labeling was more uniform across cell types (EL4 and DC2.4 cells), with 62–65% in the cytoplasm and 21–26% in the membrane. In a study of ^111^ln-oxine labeled erythrocytes of human or rat origin, 67% and 59% of activity was distributed to the cytosol, while the membranes retained 33% and 41% of activity, respectively [23]. It is possible that the slight differences in ^111^In distribution between our results and the reported studies derived from the absence of nuclei in erythrocytes. We postulate that some ^89^Zr-bound intracellular proteins exchange within the cell, changing the intracellular distribution of ^89^Zr over time. This is true in the case of membrane-bound proteins, which can be internalized. Various transport proteins, such as transferrin or signaling molecules, commonly traffic between compartments. As virtually all of the ^89^Zr-oxine bound to intracellular proteins within 5 min of incubation, ^89^Zr probably does not readily bind to proteins newly generated after labeling. Although ^89^Zr alone does not permeate the plasma membrane, it is also possible that some ^89^Zr-bound proteins are excreted from the cell via exocytosis or as components of vesicles such as exosomes.

While ^89^Zr-oxine readily permeates cells for labeling, there appears to be a saturation point, which is cell-type-dependent. We observed plateauing of activity when high doses of ^89^Zr-oxine were added to the cell. As ^89^Zr uptake is dependent on cell size and protein mass, we believe that these factors influence the saturation point. This conclusion is supported by the lower saturation point observed for the smaller Kit225K6 cells (approximately 60 kBq/10^6^ cells), as compared to EL4 and DC2.4 cells (approximately 90 kBq/10^6^ cells) that are larger and have a greater protein mass. Our in vitro retention assay suggested that labeling cells below the saturation point improves the ability of the cells to retain ^89^Zr. In addition, we and others have demonstrated that the ^89^Zr-oxine activity that does not cause radiotoxicity to the cells is approximately 37 kBq/10^6^ cells or below for many primary cell types [6,19], except for some radioresistant cells. The effects of ^89^Zr-oxine labeling on the cell viability and functions have been extensively evaluated in previous studies, as these are crucial steps for tracking cells using PET [6,14,15,16,17,18,19,20,21,31]. Keeping the specific activity of the cells below 37 kBq/10^6^ cells would ensure better retention of ^89^Zr without inducing radiotoxicity.

In support of our in vitro findings of higher protein binding and labeling stability with ^89^Zr-oxine compared to ^111^In-oxine, in vivo infusion of DCs labeled with either tracer revealed significantly higher %ID/g values for ^89^Zr activity in the liver and spleen, the primary organs to which infused DCs home, compared to ^111^In. Very similar biodistribution results have been reported, showing higher retention of ^89^Zr than ^111^In in the liver and spleen following infusion of eGFP-5T33 murine myeloma cells labeled with ^89^Zr-oxine (150 kBq/10^6^ cells) or ^111^In-oxine (340 kBq/10^6^ cells) [7]. Again, the higher activity requirement for ^111^In-oxine cell tracking using SPECT, compared to ^89^Zr-oxine that utilizes PET, may further accentuate the instability of ^111^In-oxine labeling. Additionally, several studies have reported radiotoxicity associated with^111^In-oxine cell labeling, particularly in radiosensitive cells [32,33,34]. Altogether, our findings suggest that ^89^Zr-oxine cell tracking using PET offers advantages over ^111^In-oxine SPECT imaging for detecting low concentrations of cells, with reduced risk of radiotoxicity.

This study has some limitations. First, we were not able to identify the exact intracellular proteins that bind ^89^Zr. Given the huge number of proteins present in cells, identifying ^89^Zr-bound protein(s) in electrophoreses gel samples was not possible. Our attempts to precipitate candidate proteins and examine if ^89^Zr was bound were not successful either. Second, we demonstrated that ^89^Zr-oxine cell labeling results in different subcellular distribution by cell type, and also when compared to ^111^In-oxine cell labeling, but the mechanisms causing these differences and whether these differences differentially affect cellular functions remain unknown. It is challenging to directly compare cellular functions, including trafficking, across different subcellular ^89^Zr distribution patterns in a cell type, as altering these patterns would require modifying the cells in ways that affect their functionality. One notable difference between ^89^Zr-oxine and ^111^In-oxine cell labeling is the higher nuclear localization observed with ^89^Zr. Both ^89^Zr and ^111^In emit Auger electrons capable of inducing DNA double-strand breaks [35]. Do the differences in their intracellular distributions influence the extent of DNA damage? A previous study reported that ^89^Zr-oxine labeling at 6–20 kBq/10⁶ cells did not induce significant DNA damage in human γδ T cells, whereas labeling at 50–90 kBq/10^6^ cells did [19]. The same group later showed that human white blood cells labeled with ^89^Zr-oxine (32.9 ± 9.2 kBq/10⁶ cells) and ^111^In-oxine (52.8 ± 26.1 kBq/10⁶ cells) exhibited no significant difference in DNA double-strand breaks, although a variable dose of ^111^In was used [31]. Further studies would be needed to address these questions.

In conclusion, we show that ^89^Zr-oxine cell labeling results in nearly complete protein binding in contrast to ^111^In-oxine in which only <65% is protein-bound. Protein mass and the size of cells were determining factors for the cellular incorporation of ^89^Zr. Intracellular protein binding of ^89^Zr can contribute to the stability of the label within the cell; however, this binding is saturable, and higher doses can destabilize binding. ^89^Zr-oxine labeled cells retained radioactivity at higher levels than ^111^In-oxine labeled cells following infusion to mice. These findings deepen our understanding of the mechanisms underlying ^89^Zr-oxine cell labeling and its stability both in vitro and in vivo. These characteristics of ^89^Zr-oxine support its utility for reliable long-term cell tracking with PET imaging and its clinical applications, such as monitoring cell-based therapies and investigating immune cell dynamics.

## Data Availability

All data generated in this study are included in the manuscript.

## Supplementary Materials

The following supporting information can be downloaded at: https://www.mdpi.com/article/10.3390/pharmaceutics17040518/s1, Figure S1: Subcellular fractionation determined by Western blotting; Figure S2: ^89^Zr retention was lower in cells labeled with ^89^Zr-oxine at a dose above the incorporation threshold; Table S1: ^89^Zr-oxine labeling and various cellular parameters by cell type.
