# Supplementary material for: Intracellular Protein Binding of Zr-89 Oxine Cell Labeling for PET Cell Tracking Studies
Source: Pharmaceutics. 2025 Apr 15;17(4):518. doi: 10.3390/pharmaceutics17040518 (PMC12030610; doi:10.3390/pharmaceutics17040518)
Supplement: Supplementary file 1 [file pharmaceutics-17-00518-s001.zip › pharmaceutics-3546557-supplementary.pdf]

Intracellular Protein Binding of Zr-89 Oxine Cell Labeling for PET Cell Tracking Studies

Emmanuel Nyong [1,2], Yutaka Kurebayashi [1,3], Kingsley O. Asiedu [1,4], Peter L. Choyke [1] and Noriko Sato [1,*]

[1] Molecular Imaging Branch, Center for Cancer Research, National Cancer Institute, National Institutes of Health, Bethesda, MD 20892, USA; ecnyong@utmb.edu (E.N.); y_kurebayashi@keio.jp (Y.K.); kingsley.asiedu@duke.edu (K.O.A.); pchoyke@mail.nih.gov (P.L.C.)
[2] Department of Surgery, The University of Texas Medical Branch, Galveston, TX 77555, USA
[3] Department of Pathology, Keio University School of Medicine, Tokyo 160-8582, Japan
[4] Department of Radiology, Duke University Medical Center, Durham, NC 27710, USA
[*] Correspondence: saton@mail.nih.gov; Tel.: +1-240-858-3079; Fax: +1-240-541-4526

Supplementary Figures

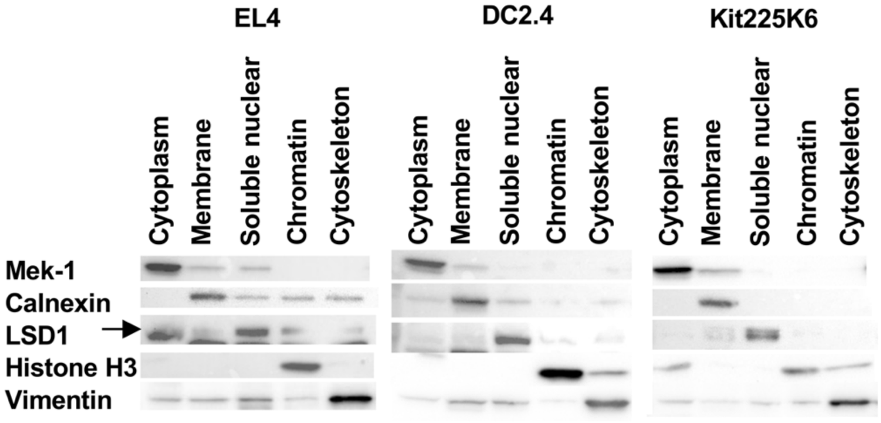

Figure S1. Subcellular fractionation determined by western blotting. Subcellular fractionated samples of ^{89}Zr-oxine labeled EL4, DC2.4 and Kit225K6 cells were subjected to western blotting. Marker proteins Mek-1, calnexin, lysine-specific demethylase 1 (LSD1), histones H3 and vimentin were probed for cytoplasmic, membrane, soluble nuclear, chromatin-bound, and cytoskeletal protein subcellular fraction, respectively (representative data of n > 3). The arrow indicates the location of LSD1 bands in the EL4 cell fractions.

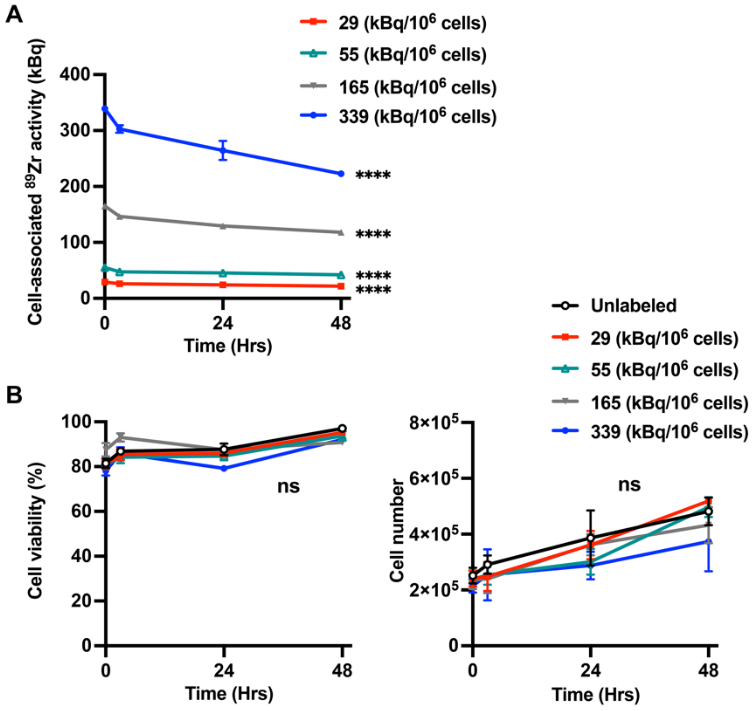

Figure S2. ^{89}Zr retention was lower in cells labeled with ^{89}Zr-oxine at a dose above the incorporation threshold. **(A)** Kit225K6 cells were labeled with ^{89}Zr-oxine at 29, 55, 165, and 339 kBq/10^6 cells and decay-corrected cell-associated activity was measured over 48 hours. The graph show the changes of ^{89}Zr activity started with 10^6 cells at 0 hr timepoint for each specific activity condition (n = 3, ****: $p < 0.0001$ in all comparisons, by a repeated measure two-way ANOVA). **(B)** During the 48 hr observation, viability (left) and number (right) of the cells did not differ among the groups, minimizing the effect of ^{89}Zr release from dead cells on the analysis of labeling dose-effects on ^{89}Zr retentions (n = 3, ns: not significant, by a repeated-measure two-way ANOVA). The data are shown as mean ± standard deviation.

Supplementary Table

Table S1. ^{89}Zr-oxine labeling and various cellular parameters by cell type.

Cell type	Incorporated ^{89}Zr (kBq/10^6 cells)	Labeling efficiency (%)	FSC (cell size)	Total protein concentration (µg/µL)	SSC (granular/ vesicular contents)
Naïve T	0.75 ± 0.06	20.76 ± 1.82	215.33 ± 2.08	1168.75 ± 225.70	29.07 ± 1.12
Kit225K6	0.92 ± 0.08	28.01 ± 0.54	320.67 ± 15.70	1417.53 ± 306.05	225.00 ± 1.00
NK	0.97 ± 0.13	28.27 ± 1.56	317.33 ± 5.13	1416.97 ± 350.93	99.00 ± 5.41
EL4	1.06 ± 0.06	31.83 ± 1.05	320.67 ± 4.73	1931.84 ± 135.14	202.67 ± 5.69
DC	1.19 ± 0.07	35.20 ± 1.70	364.33 ± 15.14	2167.52 ± 408.77	298.67 ± 18.15
Monocyte	1.33 ± 0.08	38.90 ± 2.28	391.33 ± 26.84	3580.31 ± 255.35	172.33 ± 32.52
DC2.4	1.38 ± 0.07	44.25 ± 1.70	498.00 ± 3.00	3716.73 ± 153.37	232.00 ± 10.15
MC38	1.95 ± 0.12	53.84 ± 3.65	521.33 ± 13.32	6186.37 ± 745.04	281.33 ± 15.70

The data represent mean ± standard deviation.
